# High Surface Proton Conduction in Nanostructured ZIF-8

**DOI:** 10.3390/nano9101369

**Published:** 2019-09-24

**Authors:** Daniel Muñoz-Gil, Filipe M. L. Figueiredo

**Affiliations:** Department of Materials Engineering and Ceramics, CICECO—Aveiro Institute of Materials, University of Aveiro, 3810-193 Aveiro, Portugal

**Keywords:** ZIF-8, nanostructure, surface, protonic conductivity

## Abstract

The zeolitic imidazolate framework-8 (ZIF-8) combines a significantly high microporosity with an excellent thermal, chemical, and hydrothermal stability. Here, we demonstrated that ZIF-8 can display significant levels of protonic conductivity through a water-mediated surface transport mechanism associated to the presence of di-coordinated Zn ions revealed by X-ray photoelectron spectroscopy. A set of powders with particle sizes from 2.8 µm down to 80 nm studied by dynamic water vapour sorption analysis was used to demonstrate that water adsorbs predominantly in the micropore cavities of microcrystalline ZIF-8, whereas adsorption on the external surface becomes the dominant contribution for the nanostructured material. Impedance spectroscopy in turn revealed that the protonic conductivity of the nanocrystalline ZIF-8 was two orders of magnitude higher than that of the micron-sized powders, reaching approximately 0.5 mS·cm^−1^ at 94 °C and 98% relative humidity. Simple relations were derived in order to estimate the potential gains in water uptake and conductivity as a function of the particle size. This new strategy combining particle nanostructuring with surface defects, demonstrated here for one of the most know metal organic framework, is of general application to potentially boost the conductivity of other materials avoiding chemical functionalization strategies that in most if not all cases compromise their chemical stability, particularly under high humidity and high temperature conditions.

## 1. Introduction

Over the past two decades, metal organic frameworks (MOFs) have undergone rapid development due to the fact of their designable and tuneable structures–properties, which may present a wide range of important applications like gas storage, catalysis, and energy applications. [1,2,3,4,5,6]. The use of ionic conducting MOFs in fuel cell electrolytes has also been envisaged and a number of structures have been discovered presenting technologically viable levels of protonic conductivity (>10 mS·cm^−1^) [7,8,9,10,11]. However, it is also true, and often not referred to, that most of these structures are not stable under the acidic hydrothermal medium of a proton exchange membrane fuel cells (PEMFCs), showing a tendency to hydrolyse especially when they contain acid functional groups.

The zeolitic imidazolate framework of type 8 (ZIF-8) is an organic–inorganic hybrid material with the sodalite-type structure, where Zn (or Co) metal centres are tetra-coordinated by imidazolate linkers forming a three-dimensional network of micropores with an equivalent spherical diameter of 11.6 Å connected by narrow 3.4 Å apertures [12,13,14]. This particular structure allied to the chemical composition are the reason for the exceptional thermal and hydrothermal stability, and the extraordinarily high specific surface area (typically in excess of 1500 m^2^·g^−1^) of these materials, which make them attractive in applications of storage [15,16], separation [17,18], and catalysis [19] of various chemicals.

The incorporation of a secondary phase such as a salt or phosphoric acid into the interconnected micropore structure of ZIF-8 has been explored as a way to design novel hydroxyl [20] or proton [21] conductors, respectively, which are expected to maintain the chemical and thermal stability of the host structure. Using water as a secondary phase would make ZIF-8 a water-mediated proton conductor, but the hydrophobic character of the material [22,23,24] leads to ionic conductivity well below 1 mS·cm^−1^ even at 98% relative humidity (RH) and approximately 100 °C [25]. Chemical modification of the inner surface with, for example, acidic functional groups appears as an obvious strategy to increase the water uptake of the material, but the chemical stability is likely to be severely compromised [26]. The design of MOFs with a hierarchical micro- and mesopore structure has recently been proposed to achieve proton transport using only changes in water vapour pressure, although the demonstrated conductivity levels are rather low (less than 0.1 mS·cm^−1^) [10].

It is well known that the composition of the surface is usually different from the composition of the bulk. Recent experimental results confirm the importance of the external surface composition for an important number of reactions in ZIF materials [16,17,18]. Ke Zhang et al. [24] reported that the ZIF-8 shows an exceptionally large inner surface area and pore volume of micropores. However, they found that the water uptake capacity can be improved more than two times when decreasing the particle size of the ZIF-8 from 324 to 0.4 µm. This indicates that the water sorption is closely connected with the outer surface properties [24]. The behaviour of the external surface of ZIF-8 could be due to the different particle sizes but also to the variable coordination environment of the zinc nucleus, which could affect the water sorption behaviour. This may occur because Zn with low coordination is expected to be preferentially located on the surface, where it is coordinated by hydroxyl groups. Water molecules can easily form hydrogen bounds with these surface hydroxyl groups, thus improving the water sorption capacity in comparison to a defect-free surface [22,23,24]. Celine Chizallet et al. [27] reported that at room temperature and atmospheric pressure, tri-(Zn–Im_3_) and di-(Zn–Im_2_) coordinated zinc ions (by imidazolate linkers) are the most stable terminations, both having water adsorption capacity but with the di-coordinated species being most reactive [27]. Regarding water uptake, the most preferred adsorption mode in both terminations is molecular adsorption rather than dissociation [28].

Here, one explores the potential of nanostructuring in order to induce extensive formation of hydrophilic defects on the surface of ZIF-8, as yet another approach to enhance the proton transport ability of a chemically stable MOF. This study was based on a set of ZIF-8 powders with average particle size of 80 nm, 1.1 µm and 2.8 µm, which are characterized by a battery of methods enabling the assessment of the relationships between the particle morphology/microstructure, the surface structure/composition, the water sorption behaviour, and the ionic conductivity behaviour under variable temperature and relative humidity.

## 2. Materials and Methods

### 2.1. Materials

Zinc nitrate hexahydrate (99%), (Zn(NO_3_)_2_·6H_2_O) 2-Methylimidazole (2-MeIm, 97%), methanol (99.8%), and sodium formate (NaHCO_2_, 99%) were purchased from Alfa Aesar (Thermo Fisher Scientific Inc., Kandel, Germany) and used without further purification.

### 2.2. Synthesis of ZIF-8A (~80 nm)

The nanocrystal was synthesized as reported in our previous work [25]. The synthesis starts with the preparation of two solutions, one by dissolving 0.002 mol of zinc nitrate hexahydrate (Zn(NO_3_)_2_·6H_2_O) in 50 mL of methanol, and another containing 0.016 mol of 2-MeIm in the same amount of methanol. The solutions were left to stand for 1 h at room temperature and then the 2-MeIm solution was poured into the (Zn(NO_3_)_2_·6H_2_O) solution under stirring. The formed nanocrystals were then separated from the milky suspension by three cycles of centrifugation (6000 rpm, 30 min) and washed with methanol under ultrasounds. The Zn:2-MeIm:MeOH molar ratio was 1:8:1000.

### 2.3. Synthesis of Micro-Sized ZIFs-8

#### 2.3.1. Synthesis ZIF-8B (~1.1 µm)

The ZIF-8 crystals were synthesized by modifying the procedure reported by C. Zhang and co-workers [29]. A solution of Zn(NO_3_)_2_·6H_2_O (1.7643 g) in 60 mL of methanol and a second solution of 2-MeIm (1.9480 g) and NaHCO_2_ (0.4037 g) in another 60 mL of MeOH were prepared. The latter solution was poured into the former solution and stirred until mixed. The molar ratio of Zn:2-MeIm:NaHCO_2_:MeOH was 1:4:1:500. The solution was left for 12 h in a sealed glass jar at room temperature. The product was collected after three centrifugation cycles at 6000 rpm, each for 30 min, and subsequently washed with MeOH.

#### 2.3.2. Synthesis ZIF-8C (~2.8 µm)

The largest ZIF-8 crystals were also synthesized by modifying the procedure reported by C. Zhang and co-workers [29]. The synthetic procedure was the same as that used for ZIF-8B, except for the lower amount of methanol. In this case, 1.7643 g of Zn(NO_3_)_2_·6H_2_O was dissolved in 36 mL methanol, and 1.9480 g of 2-MeIm and 0.4037 g NaHCO_2_ were dissolved in 36 mL methanol, so that the molar ratio Zn:2-MeIm:NaHCO_2_:MeOH was 1:4:1:300.

### 2.4. Characterization Techniques

The identification of the crystalline phase was carried out by powder X-ray diffraction (XRD) using an Empyrean powder diffractometer with Cu Kα_1_ (λ = 1.5406 Å) at room temperature. The patterns were taken in step mode with a step size equal to 0.013 (2*θ* degrees) and time per step equal to 39 s. The XRD patterns were also collected under variable temperature using a Panalytical X’PERT Pro3 diffractometer (Malvern Panalytical, Malvern, UK). LeBail fitting [30] of the XRD patterns was performed to obtain the lattice parameters using the FullProf Suite (version June-2015 Copyleft (c), JGP-JRC) [31]. The average crystallite diameter was determined from these data using the Scherrer equation.

Scanning electron microscopy (SEM) (HITACHI SU-70, Hitachi, Ltd. Tokyo, Japan), coupled with energy-dispersive spectroscopy (EDS, Bruker Quantax 400, Bruker AXS Microanalysis, Berlin, Germany) was used to study the morphology and elemental composition for each sample. For the analysis, the powder was dispersed in methanol at 10 ppm and then suspended on a copper-supported carbon film. The average Feret diameter of the particles (*D*) was determined from the SEM micrographs using Image J (v1.52a).

The nitrogen sorption isotherms were obtained on a Micromeritics Tristar 3000 (Micromeritics Instrument Corp., Norcross, GA, USA) porosimeter at 77 K, degassing the samples overnight before the measurements, at 423 K and 10 mPa. The specific surface area (*S*_BET-N2_) of the materials was determined through the Brunauer–Emmett–Teller equation. The external surface area (*S*_ext_) for each sample was calculated by *S*_ext_ = 6/*Dρ*, where *D* is the equivalent spherical diameter (determined as the Feret diameter) of the particle and *ρ* is the crystal density derived from the unit cell volume.

The water vapour sorption isotherms were measured on a DVS (Dynamic Vapour Sorption) device from Surface Measurements Systems (London, UK) with 200 sscm of N_2_ as carrier gas. The data were collected for sorption and desorption cycles using as stability criterion a minimum for the variation of the mass over time (d*m*/d*t*) of 0.002% min^−1^. When the d*m*/d*t* minimum was not attained (observed only for 98% RH), the maximum stage time at each humidity was 360 min. Prior to the measurements, the samples were preheated in situ at 150 °C for 2 h at 0% RH and then cooled down to the sorption temperature, always under 0% RH. The Brunauer–Emmett–Teller equation was also used to obtain estimates of the specific surface area (*S*_BET–H2O_) from the water isotherms.

The surface composition was studied by X-ray photoelectron spectroscopy (XPS). The spectra were acquired in an ultra-high vacuum system with a base pressure of 0.02 µPa. The XPS system was composed of three components: a hemispherical electron energy analyser (Phoibos 150, SPECS Surface Nano Analysis GmbH., Berlin, Germany), a delay-line detector and a monochromatic AlKα (1486.74 eV) X-ray source. High-resolution spectra were recorded at a normal emission take-off angle and with a pass-energy of 20 eV, which provided an overall instrumental peak broadening of 0.5 eV. Sample preparation consisted of dilution in ultrapure water and deposition on a silicon substrate by a drop-coating technique.

The protonic conductivity (*σ*) was measured by impedance spectroscopy. Disk-shaped pellets were prepared by uniaxial pressing of the powder at 1.53 MPa and then isostatically at 200 MPa. Silver electrodes were applied on both sides of the pellets (Agar Scientific, Ltd., Stansted, UK). The samples were placed on ceramic tubular sample-holders inside a climatic chamber (ACS DY110, Angelantoni Test Technologies Srl., Massa Martana, Italy) in order to carry out the measurements under variable temperature (40–94 °C) and relative humidity (RH, 20–98%). Before the measurements, the pellets were left overnight at 120 °C and nearly 0% RH. Platinum wires were used to ensure a good current collection. The impedance spectra were collected between 20 Hz and 2 MHz and variable test signal amplitude from 0.1 to 1 V, using the Agilent E4980A precision LCR meter (Agilent Technologies, Santa Clara, CA, USA). ZView software V3.5c (Scribner Associates, Inc., Souther Pines, NC, USA) was used to obtain the electrical resistance of the samples (*R*). The conductivity was calculated through *σ* = *L*(*RA*)^−1^, where L is the thickness of the pellets and A is the surface area of the electrodes

## 3. Results

### 3.1. Structure and Microstructure

**Figure 1:**
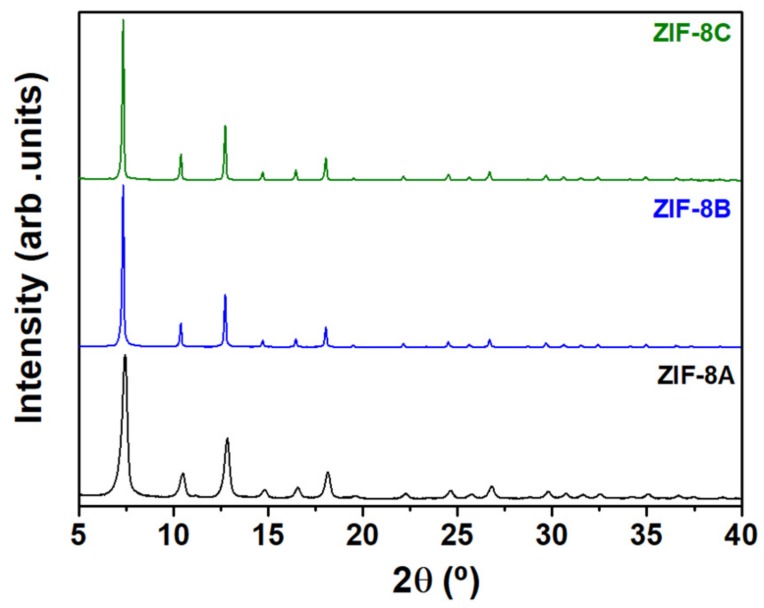
Room temperature XRD patterns of ZIF-8A (black), ZIF-8B (blue) and ZIF-8C (green). Profile fitting was used to index the XRD patterns on the cubic space group I-43m.

Figure 1 shows the room temperature XRD patterns of ZIF-8 samples prepared under different synthesis conditions. The three compounds are single phase and the patterns can be indexed using a cubic system with space group I-43m demonstrating the typical sodalite (SOD) zeolite-type structure (Figure S1), in agreement with others [12,25] and confirming that ZIF-8 was successfully synthesized. Simple visual inspection of the patterns showed clearly broader reflections for ZIF-8A, indicating a distinctively smaller crystallite size.

Table 1 shows a slight increase (less than 0.5%) in the lattice parameters with decreasing particle size and/or with the increase of the imidazole content. One may link this trend to the solvent content, which is the only parameter that varies systematically across the synthesis of the three materials (the MeOH content decreases with increasing particle size and, thus, with decreasing lattice parameter). Any further explanation of this correlation would be speculative without further work, and not necessary as it does not affect the subsequent discussion and conclusions.

**Figure 2:**
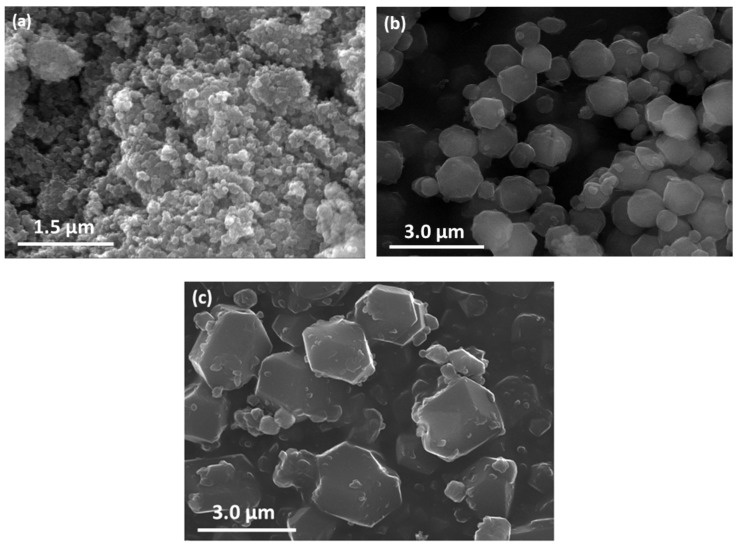
SEM images of (**a**) ZIF-8A, (**b**) ZIF-8B, and (**c**) ZIF-8C.

The crystal morphology of ZIF-8 compounds was studied using a scanning electron microscope (SEM), as summarized in Figure 2. The synthesis conditions have an important impact on the average particle size, with the largest particle size obtained using the smallest Zn:2-MeIm molar ratio, whereas the nanopowder was obtained when 2-MeIm excess was used. This effect could be explained due to the decreasing number of deprotonated ions for coordination with Zn^2+^, which may drive to smaller nucleation rate and consequently higher crystal sizes. The solvent content also has an impact on the crystal size. The MeOH induces the dissociation of the amine hydrogen of 2-MeIm, which coordinates with the Zn^2+^. Therefore, a higher amount of solvent promotes dissociation of more 2-MeIm, hence facilitating coordination between the zinc and the nitrogen atoms, increasing the nucleation rate and, consequently, leading to smaller crystal sizes.

The hydrothermal stability of the materials was assessed by submitting the powders to an atmosphere saturated with 98% relative humidity at 94 °C and 98% during 15 h. No signs of decomposition could be detected, notably by XRD, where only the reflections corresponding to ZIF-8 could be detected in the diffractograms (Figure S2). Also, no important changes in the lattice parameter were observed. This apparent stability of the samples is also supported by the impedance measurements, discussed in more detail in the electrical conductivity section of this paper. In fact, if any substantial dissolution or decomposition of the material would indeed occur under high temperature and humidity, this would likely affect the interparticle connectivity of the pellet and, thus, increase the pellet impedance over the time of the measurement, which did not occur—actually the opposite was observed. Nevertheless, future studies are planned to provide a deeper assessment of the stability of the material in harsh hydrothermal conditions, namely, by using complementary methods such as mass or atomic absorption spectroscopy and ionic chromatography for the metal, and GC/MS, HPLC or NMR for the ligand.

### 3.2. Sorption Isotherms

The N_2_ adsorption isotherms are of type I for all ZIF-8, thus confirming the presence of micropores (Figure S3). The nanostructured sample shows, in addition, a small hysteresis due to the presence of some mesoporosity among the agglomerated nanoparticles. Brunauer–Emmett–Teller (BET) analysis results given in Table 1 show, as expected, that increasing particle size decreases the specific surface area (*S*_BET–N_2__). At first, this decrease may be attributed to the lower contribution of the external surface area (*S*_ext_) of the larger particles. Indeed, the difference in *S*_ext_ obtained for ZIF-8A and ZIF-8B (212 m^2^∙g^−1^) explains, to a large extent, the difference in S_BET–N_2__ for these same two samples, which was about 180 m^2^·g^−1^ (see Table 1). However, the *S*_BET–N_2__ of ZIF-8C (the sample with coarser particles) was much lower than for ZIF-8B, with the difference exceeding by far the differences in *S*_ext_. One possible explanation for the lower inner surface was the presence of a higher content of solvent molecules in the larger particles of ZIF-8C.

**Figure 3:**
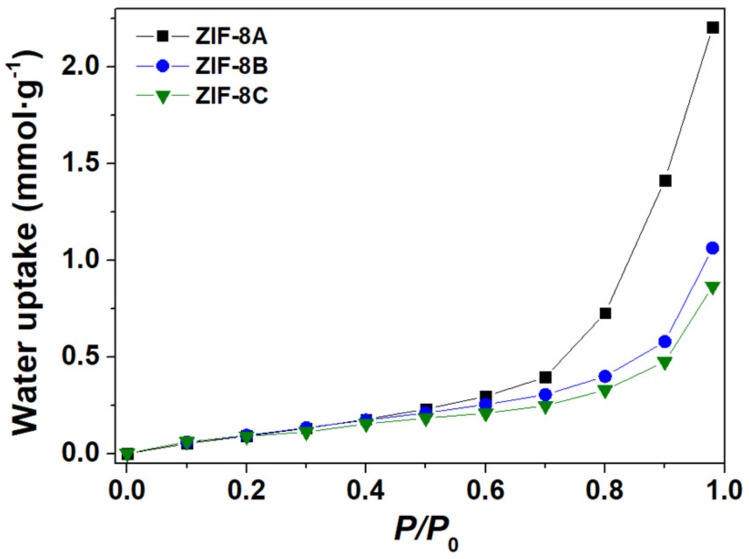
Water adsorption isotherms measured at 30 °C for ZIF-8A (black), ZIF-8B (blue) and ZIF-8C (green).

The water adsorption isotherms obtained at 30 °C are depicted in Figure 3 for the three materials (equivalent data collected at 60 °C shown in Figure S4). At low humidity (roughly *P*/*P*_0_ lower than 0.6), all ZIFs-8 samples exhibited very low water uptake which demonstrates strong hydrophobic character, regardless of the particle size, in good quantitative agreement with values reported for microcrystalline ZIF-8 [22,23,24]. At high humidity, say *P*/*P*_0_ larger than 0.6, one observes a distinct increase of the water absorption with decreasing particle size, noticed particularly for the nanostructured ZIF-8A.

The combination of the data collected at difference temperature allows the determination of the isosteric heat of adsorption using the Clausius−Clapeyron [32]. These results, depicted in Figure S5, show that the isosteric heat increases up to about 0.30 mmol of adsorbed H_2_O per gram of material, reflecting the strong hydrophobic behaviour of these materials. Above this threshold, one observes a plateau at about 40 kJ·mol^−1^ for the micro-sized samples, and 45 kJ·mol^−1^ for the nanocrystalline material. Both values are close to the molar enthalpy of water evaporation [32] (41 kJ·mol^−1^), thus suggesting a major change in the hydrophilic behaviour of the material.

The specific surface area values (*S*_BET-H_2___O_) increased from 8 m^2^·g^−1^ to 13 m^2^·g^−1^ with decreasing particle size from 2.8 μm to 80 nm. These values were substantially lower than those obtained from the N_2_ sorption data (>1400 m^2^·g^−1^, see Table 1 for details), again in agreement with the highly hydrophobic behaviour of ZIF-8. We note here that the kinetic diameter of the nitrogen molecule (3.64 Å) was larger than that of water (2.65 Å), as well as the cross-sectional area (~16 Å^2^ vs. ~11 Å^2^) [33]. This means that at least a fraction of the water may adsorb inside the pores, as it happens with the larger N_2_ molecules. This is actually confirmed for the two samples with coarser particles where, and analogously to the preceding comment on *S*_BET–N_2__, the corresponding *S*_BET–H_2___O_ is larger than *S*_ext_. However, such rationale cannot be used in the case of the nanostructured powder, where *S*_BET–H_2___O_ is much smaller than *S*_ext_ (14 m^2^·g^−1^ vs. 219 m^2^·g^−1^).

**Figure 4:**
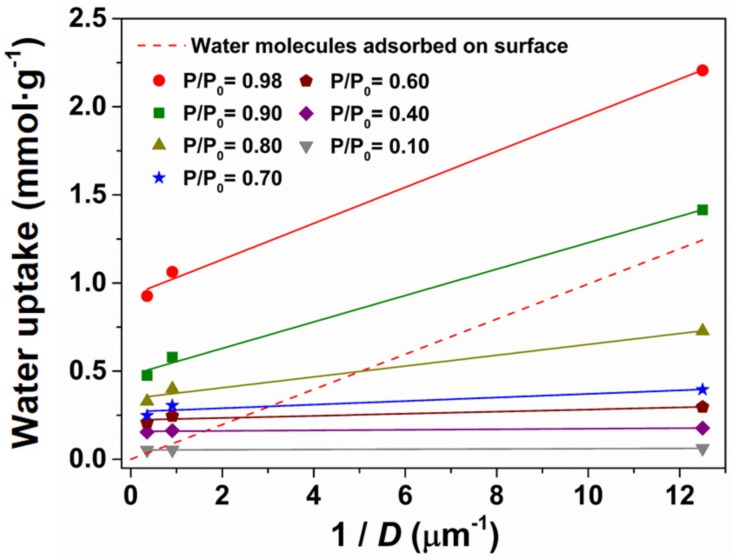
Water uptake at variable *P/P_0_* plotted as a function of the particle size of ZIF-8, compared to a prediction assuming a monolayer of water covering the external particle surface (dashed line).

The question thus remains whether water is adsorbing fully inside the cavities or on the outer surface of the particles. This can be ascertained by the plot of the water uptake as a function of the inverse of the equivalent spherical diameter of the particles (*D*), shown in Figure 4, for variable *P*/*P*_0_.

It can be seen that the water uptake was little affected by the particle size up to *P*/*P*_0_ = 0.6. This unambiguously indicates that the water was adsorbing predominately inside the pores in the three materials, since there was no effect of the surface area. However, for *P*/*P*_0_ higher than 0.7 there was a clear increase of the water uptake with decreasing particle size, which gains magnitude with increasing humidity. The adsorption of water on the external surface of the ZIF-8 particles was the only explanation for such effect. A similar trend was reported by Zhang et al. [24], but for larger particles (in the range 0.4 to 324 µm).

The number of water molecules adsorbed per g of ZIF-8 ($n_{H_{2}O}$) thus comprises those in the interior porous cavities ($n_{H_{2}O}^{\mathrm{int}}$) and those on the outer surface ($n_{H_{2}O}^{S}$), such that:

(1) $$n_{H_{2}O}=n_{H_{2}O}^{\mathrm{int}}+n_{H_{2}O}^{S}$$

Assuming full coverage of the surface by a monolayer of water molecules with spherical shape, $n_{H_{2}O}^{S}$ can be calculated taking into account the diameter of the particles (*D*) by:

(2) $$n_{H_{2}O}^{S}=6{\left(Dd\alpha_{H_{2}O}N_{A}\right)}^{-1}$$

where *d* = 0.91 g·cm^−3^ is the ZIF-8 density estimated from the unit cell volume and the molecular formula, $\alpha_{H_{2}O}$ = 1.1 × 10^−15^ cm^2^ is the cross-sectional area of a water molecule [33], and *N*_A_ = 6.022 × 10^23^ mol^−1^ is the Avogrado constant. These $n_{H_{2}O}^{S}$ estimates were plotted as a function of *D*^−1^ in Figure 4 (dashed line). Discarding the obvious off-set at the origin, the slope of $n_{H_{2}O}^{S}$ vs. *D*^−1^ was in excellent agreement with the water uptake versus *D*^−1^ data obtained at nearly saturated conditions (*P*/*P*_0_ = 0.98). Therefore, assuming that the three materials adsorb basically the same amount of water inside the pores (e.g., $n_{H_{2}O}^{\mathrm{int}}$ ≈ 1 mmol·g^−1^ at *P*/*P*_0_ = 0.98), one can estimate (for the same *P*/*P*_0_) $n_{H_{2}O}^{S}$ ≈ 1.2 mmol·g^−1^ for the nanocrystalline ZIF-8A (equal to the difference between $n_{H_{2}O}$ = 2.2 mmol·g^−1^ and $n_{H_{2}O}^{\mathrm{int}}$). This therefore suggests a considerably different type of hydration behaviour of the surface with respect to the bulk cavities of ZIF-8, which are strongly hydrophobic. One notes in this regard that at *P*/*P*_0_ = 0.98 the volume of water molecules corresponding to $n_{H_{2}O}^{\mathrm{int}}$ ≈ 1 mmol·g^−1^ corresponds to less than 0.3% of the cavity volume in ZIF-8, or less than 0.1% at *P*/*P*_0_ = 0.7.

Combining Equations (1) and (2) gives the straightforward relation:

(3) $$n_{H_{2}O}=n_{H_{2}O}^{\mathrm{int}}+6{\left(d\alpha_{H_{2}O}N_{A}\right)}^{-1}\times D^{-1}$$

which can be used to fit the data in Figure 4 in order to estimate $n_{H_{2}O}^{\mathrm{int}}$ and $n_{H_{2}O}^{S}$ as a function of *D*, for the various humidity conditions. The fitting results are listed in Table S1. Since *P*/*P*_0_ = 0.98 corresponds to full coverage of the particles external surface by water molecules, the ratio ${\left(n_{H_{2}O}^{S}D^{-1}\right)}_{P/P_{0}}:{\left(n_{H_{2}O}^{S}D^{-1}\right)}_{P/P_{0}=0.98}$ yields about 10% surface coverage at *P*/*P*_0_ = 0.7, or 30% at *P*/*P*_0_ = 0.8. This suggests that a significant amount of water may be adsorbed by ZIF-8 if one can decrease the particle size into the nanometre range (below 100 nm). Indeed, the fitting parameters in Table S1 can be readily used to predict $n_{H_{2}O}^{S}$ as a function of humidity for a variable particle size, as shown in Figure S6. These predictions show that a hypothetic ZIF-8 nanostructured to 10 nm will be able to adsorb as much water at 70% relative humidity as the ZIF-8A sample at 98%. Quite spectacularly, this 10 nm ZIF-8 can potentially adsorb approximately 10 mmol·g^−1^ in nearly saturated conditions. Both values underlie the potential of the nanostructuring approach to induce major changes in the hydrophilicity of ZIF-8, thus avoiding functionalization strategies with, for example, acidic groups that may compromise the chemical stability. One property that immediately benefits from this change is the water-mediated ionic conductivity, as shown in the following section.

### 3.3. Ionic Conductivity

**Figure 5:**
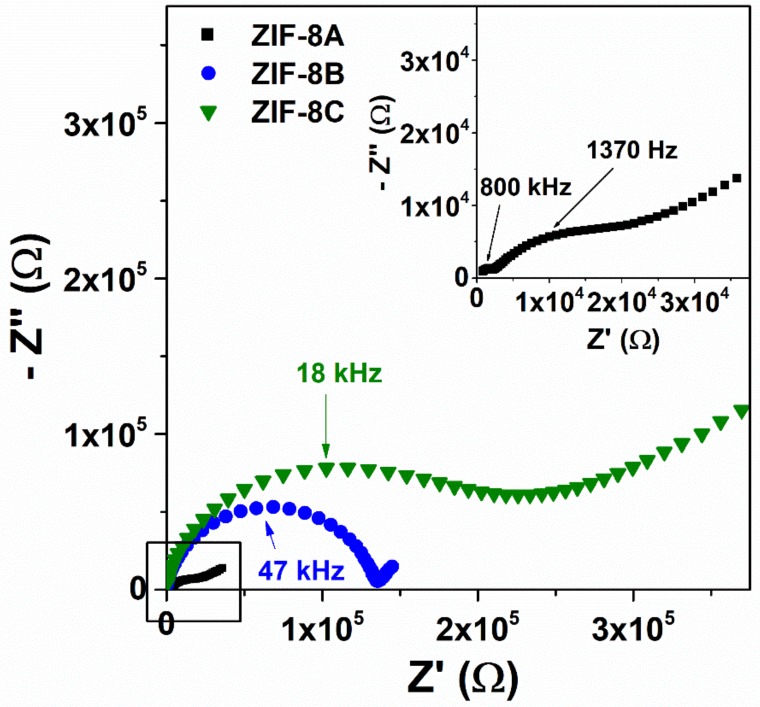
Nyquist plots collected at *P/P*_0_ = 0.98 and 94 °C for ZIF-8A (black squares), ZIF-8B (blue circles) and ZIF-8C (green triangles). The numbers indicate the peak frequency.

Impedance spectroscopy measurements were carried out under variable relative humidity (RH) and temperature to elucidate the relationship between particle size and the ionic conductivity. The Nyquist plots of micron-sized powders were composed of a regular semicircle and a capacitive tail at any temperatures and all ranges of RH. Examples of such spectra are shown in Figure S7c,d,e in the Supplementary Materials. The impedance was strongly temperature and humidity dependent for all ZIF-8, decreasing by more than four orders of magnitude between 40% and 98% RH, at 94 °C for ZIF-B (Figure S7c and decreasing by more than two orders of magnitude between 60 and 94 °C, at 98% RH for ZIF-C (Figure S7e). These results indicate that the conductivity was essentially due to the ionic transport along adsorbed water molecules, despite the hydrophobicity of the materials [22,23,24]. In most cases, the ohmic resistance of the samples used to calculate the conductivity values corresponded to the amplitude of the sole semicircle in the impedance spectrum. The impedance of the ZIF-8A nanopowder was equally dependent on RH and temperature, but the shape of the spectra changed dramatically when approaching saturated RH conditions (Figure S7b). Regardless of the temperature, these spectra show a well-defined semicircle at high frequency, followed by a depressed semicircle at intermediate frequencies and a capacitive tail at low frequency (see Figure 5 inset).

As noticed and explained on a previous study [25], one finds that both the intermediate semicircle and the capacitive tail are strongly dependent on the test signal amplitude. This indicates that these impedance contributions are of interfacial nature and is the amplitude of the high-frequency semicircle that corresponds to the sample ohmic resistance. This could be obtained by least squares fitting the spectrum to an equivalent circuit comprising two parallel RC elements in a series, and assuming the usual constant phase element to account for the abatement of the arcs [25].

**Figure 6:**
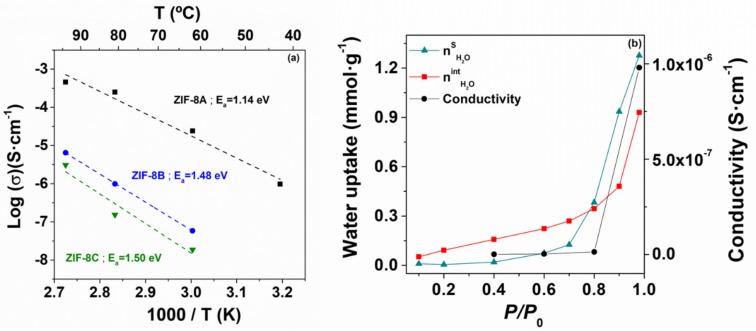
(**a**) Log-scaled protonic conductivity of ZIF-8A (black), ZIF-8B (blue) and ZIF-C (green), plotted as a function of the inverse temperature and measured at 98% relative humidity; (**b**) Conductivity, $n_{H_{2}O}^{S}$ and $n_{H_{2}O}^{int}$ as a function of relative humidity for the nanostructured ZIF-8A.

Figure 6a displays the temperature dependence of the ionic conductivity at 98% RH for the set of ZIF-8 samples. Closely following the water uptake capacity, the conductivity increased with decreasing particle size. The effect was particularly noticeable for the nanocrystalline ZIF-8, where the maximum conductivity (approximately 0.5 mS·cm^−1^ at 94 °C and 98% RH) was about two orders of magnitude higher than that of the micron-sized powders. On a first approximation, one may correlate the conductivity difference among the three ZIF-8 with the differences in the water uptake, which was higher for smaller particle size (Figure 3). However, the water uptake of ZIF-8A was just about the double of that of ZIF-8B or ZIF-8C, which suggests that the observed two orders of magnitude enhancement in conductivity was not only due to the fact of an enhanced concentration of the charge carriers (water molecules), but also to enhanced mobility, denoting the onset of a different transport mechanism. In fact, the much lower activation energy observed for ZIF-8A seems to support the latter hypothesis (1.14 eV against 1.5 eV observed for both micrometric powders).

These are abnormally high-activation energies for water-mediated proton conducting MOFs, which typically lie in the range 0.1–0.6 eV [34]. Yet, they are not uncommon for MOF-impregnated systems, such as proton conducting 1H-1,2,4-triazole weak electrolyte (*E_a_* ≈ 1.8 eV) [35], or hydroxyl conducting immobilized tetrabutylammonium salt (*E*_a_ ≈ 0.7 eV) [20]. Such high-activation energies for ion transport in microporous materials like ZIF-8 is thus likely to be related to the resistance offered by the narrow opening connecting the structural cavities (3.4 Å in the case of ZIF-8). As showed in the study of the water uptake as a function of the particle size, most of the water uptake of micro-structured ZIF-8B and ZIF-8C is due to the water sorption inside the pores, i.e., $n_{H_{2}O}=n_{H_{2}O}^{\mathrm{int}}$, resulting in a high-activation energy for ion transport, whereas the much higher surface area of ZIF-8C more than doubles $n_{H_{2}O}$ due to the surface contribution ($n_{H_{2}O}=2.2\times n_{H_{2}O}^{\mathrm{int}}$). The correlation between the boost in conductivity and the contribution of surface adsorbed water was also explicit in Figure 6b, where a relative humidity of 80% emerges as the threshold for a dominating surface contribution. Such surface transport mechanism can explain the lower activation energy for ZIF-8A. This active surface was also likely to facilitate water incorporation into the structure decreasing the interfacial impedance, which become apparent in the impedance spectra of the H_2_O, Ag(s)|ZIF-8A|Ag(s), H_2_O cell used for the conductivity measurements at high relative humidity (see Figure 5, inset).

The extraordinary difference in hydrophilicity between the bulk and the surface of ZIF-8 particles must be related to differences on the composition and/or surface structure. The following section of the paper attempts a characterization of those differences by X-ray photoelectron spectroscopy (XPS).

### 3.4. Analysis of Surface Composition by XPS

The samples with finer (ZIF-8A) and coarser (ZIF-8C) particle size were studied by XPS. While XPS is not strictly a surface characterization technique since it gathers information from up to 10 nm from the surface (as opposed to secondary ion mass spectroscopy, SIMS, or low-energy ion scattering spectroscopy, LEIS), the large difference in the external surface area between both samples ensures that the surface contributions will be much larger in the case of ZIF-8A and, thus, the differences in the results of nano- and microstructured samples should be meaningful.

In the ideal ZIF-8 structure, Zn is tetrahedrally coordinated by four imidazole ligands. However, Zn ions with lower coordination are likely to form on the surface introducing defects (active sites) allowing hydrogen bonding with vapour-phase water, thus improving the water absorption on the surface. Celine Chizallet et al. [27,28], reported that tri- (Zn–Im_3_) and di-coordinated (Zn–Im_2_) Zn ions were the most stable at room temperature and unit pressure, with both terminations showing water adsorption capacity. In addition, the Zn–Im_2_ defect shows higher reactivity than Zn–Im_3_ [27]. These types of defects can be detected by XPS.

The whole XPS spectra of both ZIF-8A and ZIF-8C show peaks corresponding to C 1s, O 1s, N 1s, and Zn 2p (Figure S8). A more detailed inspection of these data show that Zn and C environments are similar for both samples, as revealed by the high-resolution spectra depicted in Figures S9 and S10. The Zn binding-energy range detailed in Figure S9 shows peaks at 1022.3 eV (ZIF-8A) and 1022.9 eV (ZIF-8C) that correspond to Zn 2p_3/2_, and peaks at higher binding energies of 1045.4 eV (ZIF-8A) and 1045.9 eV (ZIF-8C) associated to Zn 2p_1/2_ [36,37]. The 23 eV difference between Zn 2p_3/2_ and Zn 2p_1/2_ indicates the oxidation state +2 for Zn ions in both samples [38,39]. The Zn 2p_3/2_ and Zn 2p_1/2_ binding energies for both ZIF are higher than previously reported [39,40], which may indicate the presence of low-coordination Zn atoms (i.e., zinc-rich surfaces) as reported in DFT and XPS studies of ZIF-8 [28,41].

The C 1s XPS spectra of both samples were also similar and show three different environments (Figure S10). The main peak at approximately 286 eV corresponds to the 2-methylimidazole groups and is in good agreement with previously reported XPS binding energies of other imidazole [42,43]. At higher binding energy (around 287.5 eV), the peak in both samples was consistent with the presence of carbonates [41,44]. We can also observe a carbon peak at lower binding energy, 284.6 eV, which can be due to the adventitious carbon.

**Figure 7:**
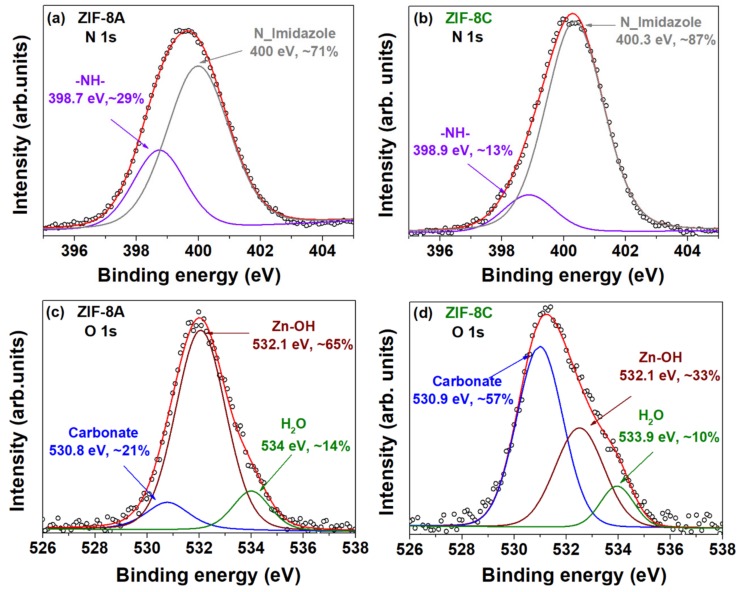
N 1s XPS spectra of (**a**) ZIF-8A and (**b**) ZIF-8C, and O 1s XPS spectra of (**c**) ZIF-8A and (**d**) ZIF-8C.

Differences among micro- and nano-sized ZIF-8 become apparent in the XPS spectra of the N 1s and O 1s regions, as depicted in Figure 7, where the peaks denoting different environments for nitrogen and oxygen atoms have different intensities. Let us start by analysing the N 1 s spectral regions shown in Figure 7a,b. Two different nitrogen atoms were found in both samples: the main peak at approximately 400 eV can be assigned to the imidazole groups, in agreement with others upon comparison with analogous compounds [42]; and another with lower binding energy (just under 399 eV), which suggests the presence of secondary amines [36,41]. The peak area of −NH− increased with decreasing particle size, which indicates a higher concentration of these secondary amines, i.e., a larger number of Zn–Im_2_ defects on the external surface of the nanostructured ZIF-8A.

The O 1s spectrum signal for both samples consisted of three peaks associated to the O–C carbonate, the Zn–OH and the water groups bound to Zn sites, respectively, characterized by binding energies around 530, 532, and 534 eV (Figure 7c,d) [43,45,46]. The peak area of Zn–OH increased with decreasing particle size, whereas the peak corresponding to carbonate decreased significantly. This again shows that the external surface area of ZIF-8A was richer in low coordination Zn than the coarser ZIF-8C, thus indicating higher water affinity for the former material. The existence of Zn–OH suggests that the formation of H_3_O^+^ protons was the likely charge-compensating species and, thus, the surface conductivity was likely to be protonic in nature.

## 4. Conclusions

In this study, we prepared a set of samples of ZIF-8 with average particle sizes of 80 nm, 1.1 µm and 2.8 µm to investigate the influence of particle size on ionic conductivity. The reduction in the particle size resulted in a huge increase in the ionic conductivity. This increase for the nanocrystalline ZIF-8 was about two orders of magnitude higher than for the micron-sized powder, reaching approximately 0.5 mS·cm^−1^ at 94 °C and 98% relative humidity. The ionic conductivity increased following closely the trend in water uptake capacity. It is suggested that for low humidity, this water is adsorbed predominately inside the pores in the three materials. However, at high partial pressures of water vapour (*P/P*_0_ > 0.7), there is a clear increase of the water uptake with decreasing particle size. This extra water at high *P/P*_0_ is adsorbed on the surface of the nanocrystalline ZIF-8 due to the higher concentration of (Zn–Im_2_) defects on the surface, as revealed by XPS, which increases the reactivity with water molecules. The surface water enables a different fast proton-conducting mechanism in nanocrystalline ZIF-8 featuring a much lower activation energy (1.14 eV) than the micron-sized samples ZIF-8 (1.5 eV), where the ionic transport occurs predominantly along the water adsorbed in the ZIF-8 structural cavities connected by narrow openings. The adopted strategy combining a nanostructured particle size with a modified defect chemistry on the surface was proved as a suitable alternative to enhance the ionic conductivity of poorly conducting, yet stable, MOF materials, thus avoiding functionalization strategies that may compromise the chemical stability of such materials.

## Figures and Tables

**Table 1:** Structural and microstructural data for the prepared zeolitic imidazolate framework-8 (ZIF-8) materials.

Sample	*D* (µm)	Lattice	Specific Surface Area (m^2^·g^−1^)		*S*_ext_ (m^2^·g^−1^)
		Parameter (Å)	*S*_BET/N_2__	*S*_BET–H_2___O_	
ZIF-8A ZIF-8B ZIF-8C	0.08 ± 0.01 1.13 ± 0.19 2.80 ± 0.71	17.0483 (2) 17.0143 (4) 16.9799 (4)	1946 1764 1450	12.8 11.4 8.11	219 6.52 2.15

## Supplementary Materials

The following are available online at https://www.mdpi.com/2079-4991/9/10/1369/s1. Figure S1: XRD patterns for ZIF-8A, ZIF-8B and ZIF-8C at room temperature. Figure S2: XRD patterns of ZIF-8A, ZIF-8B and ZIF-8C after exposure to 98% relative humidity at 94 °C, for 15 h. Figure S3: N_2_ adsorption and desorption isotherms measured at 77 K for ZIF-8A, ZIF-8B and ZIF-8C. Figure S4: Water adsorption isotherms at 60 °C for ZIF-8A, ZIF-8B and ZIF-8C. Figure S5: Isosteric heat of adsorption for ZIF-8A, ZIF-8B and ZIF-8C estimated between 30 °C and 60 °C. Figure S6: Predictions of the water uptake as a function of relative humidity at 30 °C for ZIF-8 powders with variable particle size. Figure S7: Nyquist plots collected under various temperatures and RH conditions for (a,b) ZIF-8A, (c,d) ZIF-8B and (e) ZIF-8C. Figure S8: The whole XPS spectrum for ZIF-8A and ZIF-8C. Figure S9: XPS spectra of raw data of Zn 2p of ZIF-8A and ZIF-8C. Figure S10: XPS spectra of raw data of C 1s of ZIF-8A and ZIF-8C. Table S1: Fitting result corresponding to the water uptake at variable *P*/*P*_0_ plotted as a function of the particle size of ZIF-8.
