# Supplementary material for: High Surface Proton Conduction in Nanostructured ZIF-8
Source: Nanomaterials (Basel). 2019 Sep 24;9(10):1369. doi: 10.3390/nano9101369 (PMC6835583; doi:10.3390/nano9101369)
Supplement: Supplementary file 1 [file nanomaterials-09-01369-s001.pdf]

Supporting Information

High Surface Proton Conduction in Nanostructured ZIF-8

Daniel Muñoz-Gil *and Filipe M.L. Figueiredo *

Department of Materials Engineering and Ceramics, CICECO—Aveiro Institute of Materials, University of Aveiro, 3810-193 Aveiro, Portugal, Portugal

* Correspondence: danielmg@ua.pt (D.M.G.); lebre@ua.pt (F.M.L.F.)

Received: 26 August 2019; Accepted: 19 September 2019; Published: date

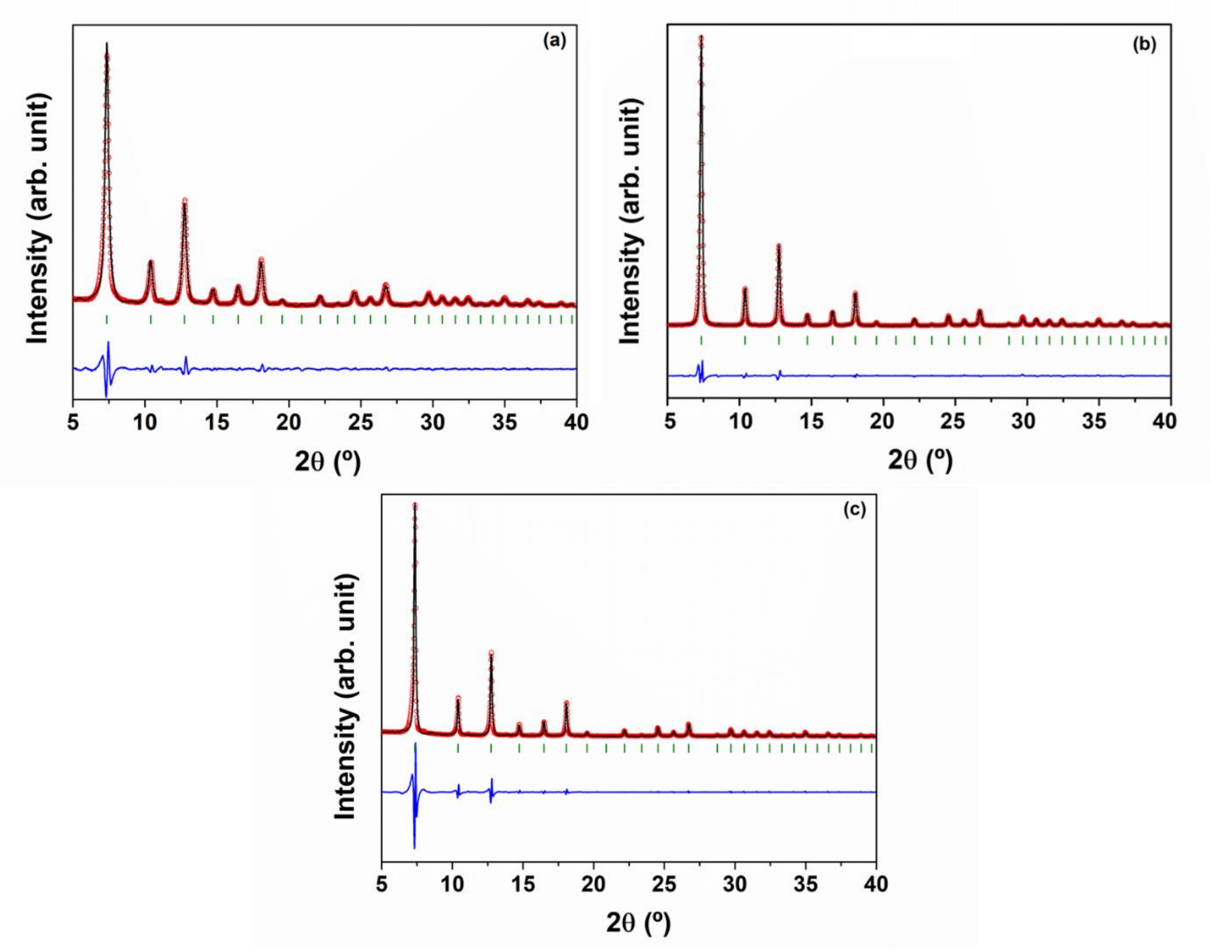

Figure S1. Experimental (red circles) and calculated (black continuous line) XRD patterns for; (**a**) ZIF-8A, (**b**) ZIF-8B and (**c**) ZIF-8C at room temperature. Profile fitting has been carried out with the I-43m space group. The vertical green bars indicate the positions of the Bragg peaks and the blue line at the bottom is the difference between the experimental and calculated patterns.

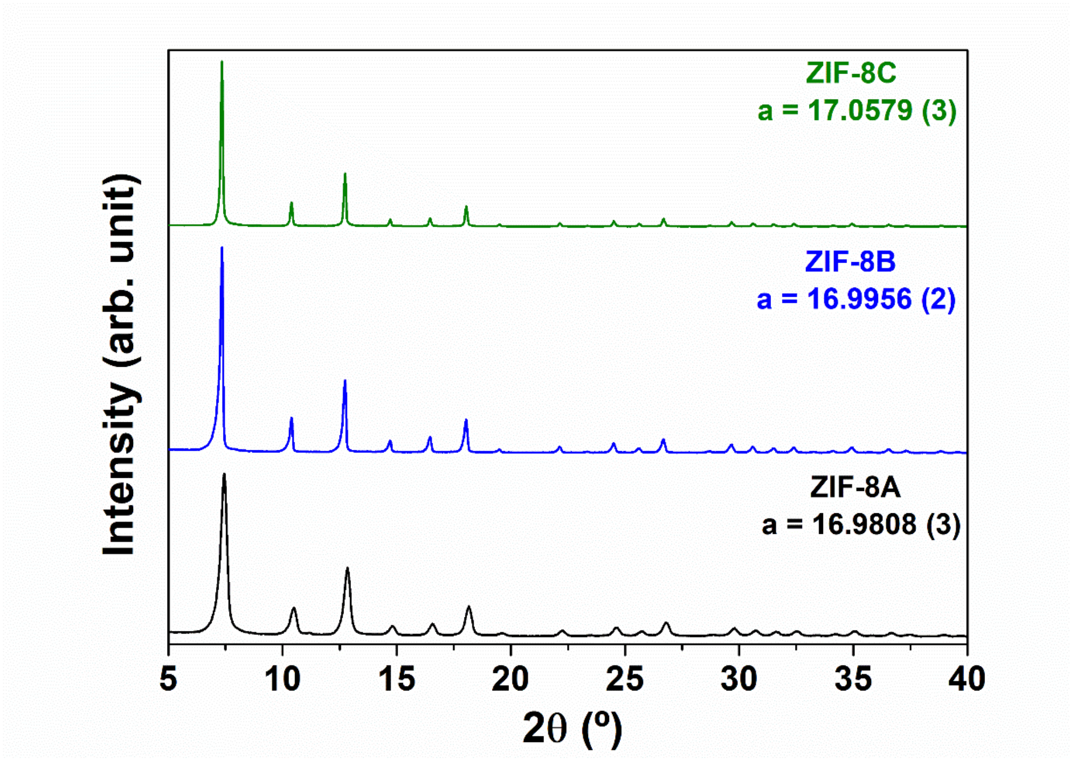

Figure S2. XRD patterns of ZIF-8A (black), ZIF-8B (blue) and ZIF-8C (green) after exposure to 98% relative humidity at 94 °C, during 15 h. Profile fitting was used to calculate the lattice parameters. The XRD patterns was indexed using a cubic system with space group I-43m

.

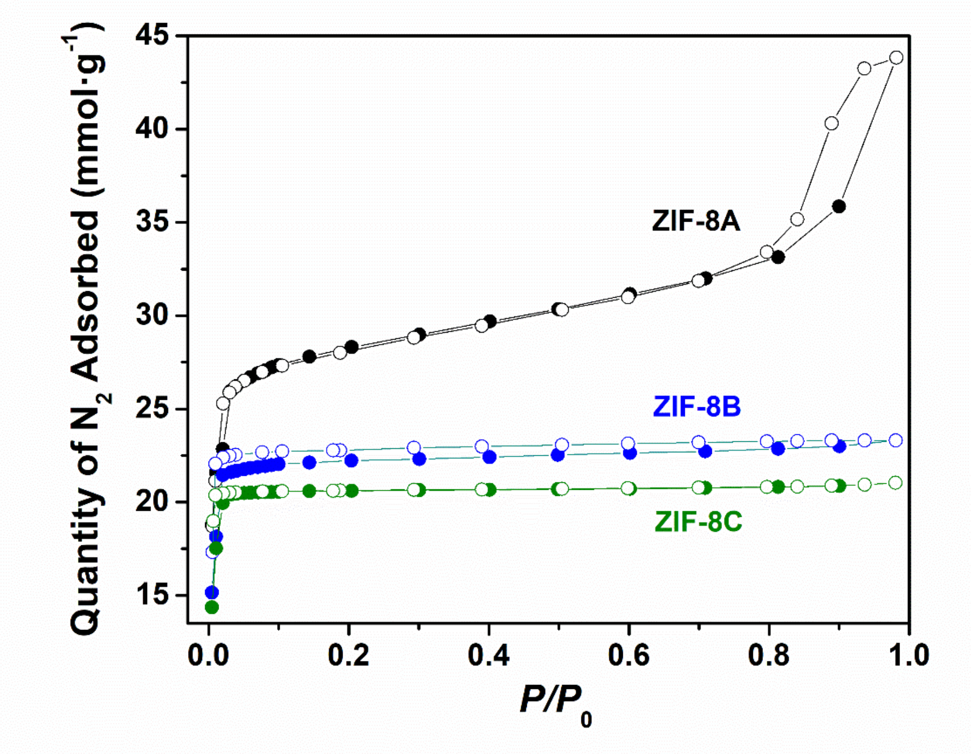

Figure S3. N_2 adsorption (solid symbols) and desorption (open symbols) isotherms measured at 77 K for ZIF-8A (black), ZIF-8B (blue) and ZIF-8C (green). The surface area was estimated using Brunauer–Emmett–Teller method.

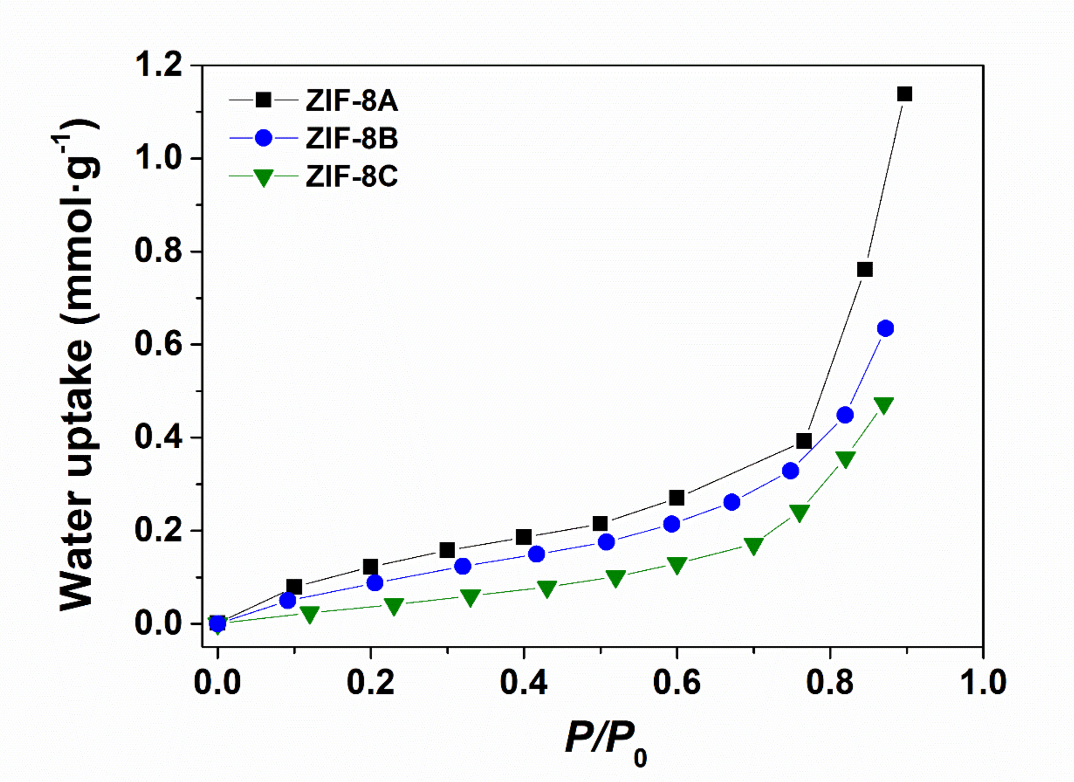

Figure S4. Water adsorption isotherms in ZIF-8A (black), ZIF-8B (blue) and ZIF-8C (green) at 60 °C.

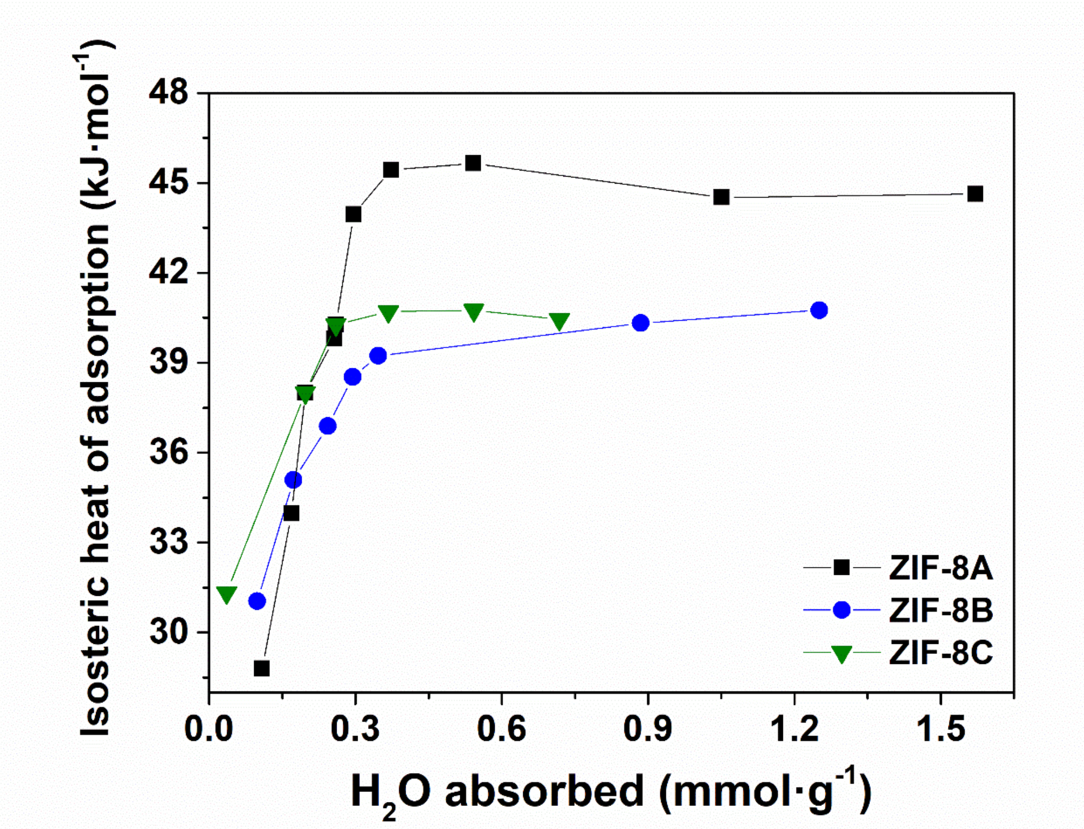

Figure S5. Isosteric heat of adsorption for ZIF-8A (black), ZIF-8B (blue) and ZIF-8C (green) estimated between 30 °C and 60 °C using the Clausius-Clapeyron equation.

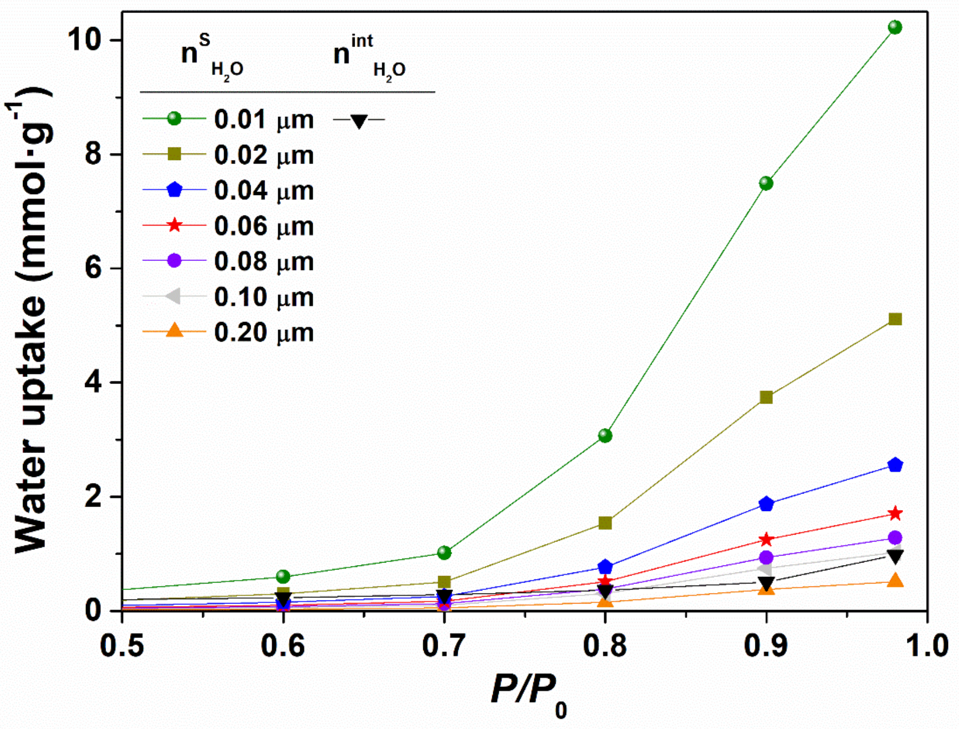

Figure S6. Predictions of the water uptake as a function of relative humidity at 30 °C for ZIF-8 powders with variable particle size, using Equation 3 and the fitting parameters in Table S1

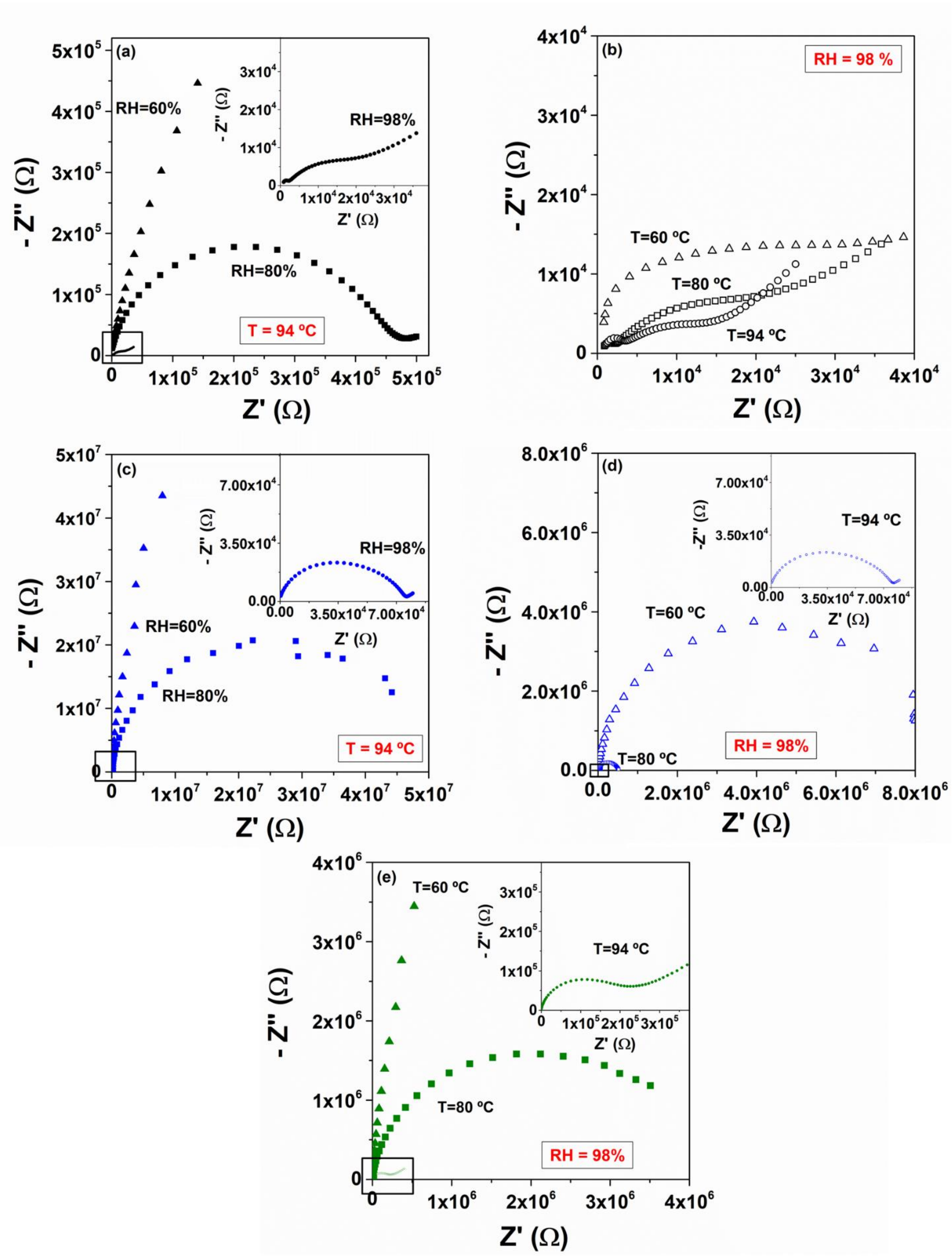

Figure S7. Nyquist plots collected under various relative humidity and temperature conditions for (**a**, **b**) ZIF-8A, (**c**, **d**) ZIF-8B and (**e**) ZIF-8C. Note that, as illustrated in (**c**), the precision of the measurement of impedance with the Agilent E4980A LCR meter decreases substantially above 10 MΩ.

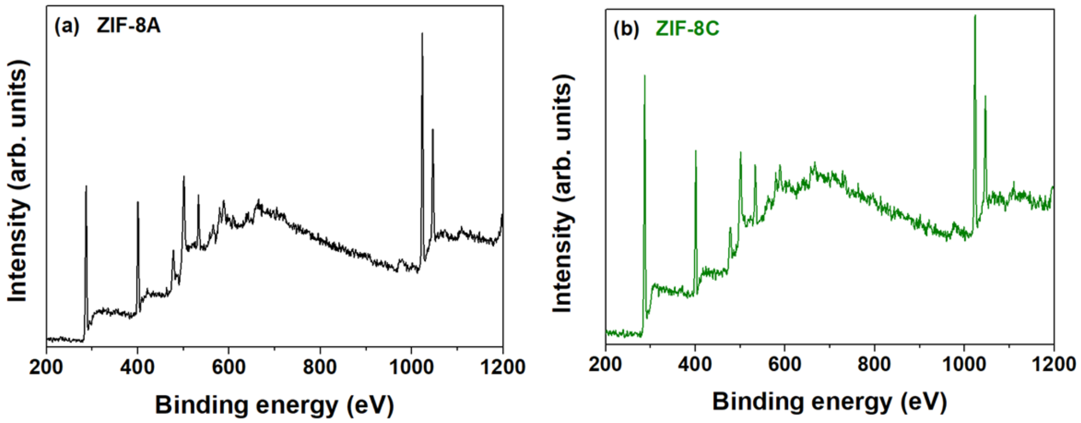

Figure S8. The whole XPS spectrum of; (**a**) ZIF-8A and (**b**) ZIF-8C.

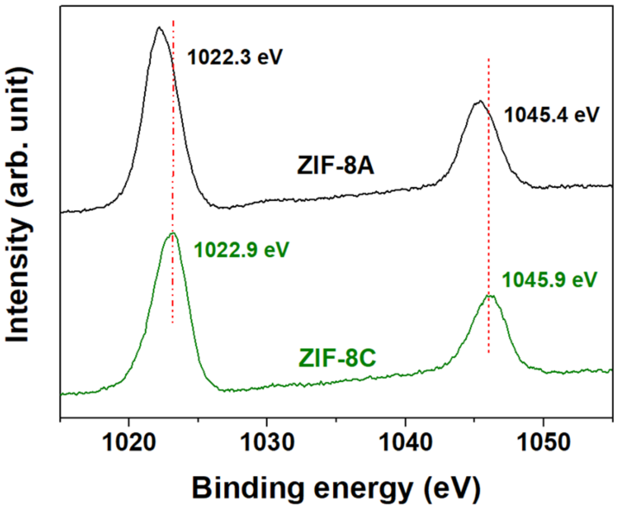

Figure S9. XPS spectra of raw data of Zn 2p of ZIF-8A (black line) and ZIF-8C (green line).

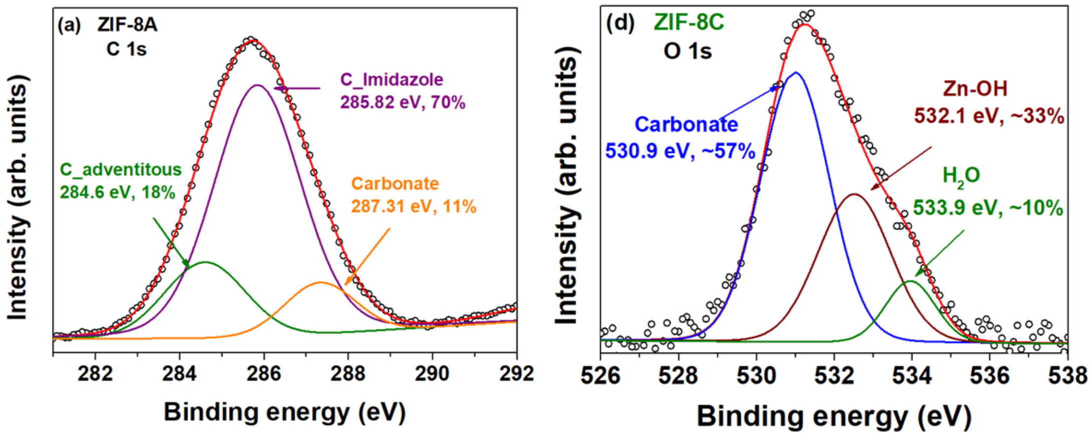

Figure S10. XPS spectra of raw data of C 1s for (**a**) ZIF-8A and (**b**) ZIF-8C.

Table S1. Results of fitting the water uptake data as a function of particle size of ZIF-8 plotted in Figure 4 to Equation (3).

P/P_0	$n^{\mathrm{int}}_{\mathrm{H_2O}}$ (mmol·g⁻¹)	$n^{\mathrm{S}}_{\mathrm{H_2O}} D^{-1}$ (mmol·g⁻¹·µm⁻¹)	r
**0.1**	$5.233\times10^{-2} \pm 8.670\times10^{-5}$	$7.497\times10^{-4} \pm 1.198\times10^{-5}$	1.000
**0.2**	$9.200\times10^{-2} \pm 2.338\times10^{-3}$	$3.765\times10^{-4} \pm 3.231\times10^{-4}$	0.759
**0.4**	$1.579\times10^{-1} \pm 3.692\times10^{-3}$	$1.541\times10^{-3} \pm 5.101\times10^{-4}$	0.949
**0.6**	$2.227\times10^{-1} \pm 1.763\times10^{-2}$	$5.960\times10^{-3} \pm 2.436\times10^{-3}$	0.926
**0.7**	$2.696\times10^{-1} \pm 2.713\times10^{-2}$	$1.013\times10^{-2} \pm 3.748\times10^{-3}$	0.938
**0.8**	$3.448\times10^{-1} \pm 2.880\times10^{-2}$	$3.070\times10^{-2} \pm 3.979\times10^{-3}$	0.992
**0.9**	$4.802\times10^{-1} \pm 3.388\times10^{-2}$	$7.489\times10^{-2} \pm 4.681\times10^{-3}$	0.998
**0.98**	$9.302\times10^{-1} \pm 4.429\times10^{-2}$	$1.023\times10^{-1} \pm 6.120\times10^{-3}$	0.998
